# Association between intraoperative end-tidal carbon dioxide and postoperative nausea and vomiting in gynecologic laparoscopic surgery

**DOI:** 10.1038/s41598-022-10727-6

**Published:** 2022-04-27

**Authors:** Li Dong, Chikashi Takeda, Hajime Yamazaki, Miho Hamada, Akiko Hirotsu, Yosuke Yamamoto, Toshiyuki Mizota

**Affiliations:** 1 Department of Healthcare Epidemiology, Graduate School of Medicine and Public Health, Kyoto University, Kyoto, Japan; 2 Department of Anaesthesia, Kyoto University Hospital, 54 Shogoin-Kawahara-cho, Kyoto, 606-8507 Japan; 3 Section of Clinical Epidemiology, Department of Community Medicine, Graduate School of Medicine, Kyoto University, Yoshida-honmachi, Sakyo-ku, Kyoto-shi, Kyoto 606-8501 Japan

**Keywords:** Outcomes research, Disease prevention, Medical research

## Abstract

Gynecologic laparoscopic surgery has a high incidence of postoperative nausea and vomiting (PONV). Studies suggest that low intraoperative end-tidal carbon dioxide (EtCO_2_) is associated with an increased incidence of PONV, but the results have not been consistent among studies. This study investigated the association between intraoperative EtCO_2_ and PONV in patients undergoing gynecologic laparoscopic surgeries under general anesthesia. This retrospective cohort study involved patients who underwent gynecologic laparoscopic surgeries under general anesthesia at Kyoto University Hospital. We defined low EtCO_2_ as a mean EtCO_2_ of < 35 mmHg. Multivariable modified Poisson regression analysis examined the association between low EtCO_2_ and PONV during postoperative two days and the postoperative length of hospital stay (PLOS). Of the 739 patients, 120 (16%) had low EtCO_2_, and 430 (58%) developed PONV during postoperative two days. There was no substantial association between low EtCO_2_ and increased incidence of PONV (adjusted risk ratio: 0.96; 95% confidence interval [CI] 0.80–1.14; *p* = 0.658). Furthermore, there was no substantial association between low EtCO_2_ and prolonged PLOS (adjusted difference in PLOS: 0.13; 95% CI − 1.00 to 1.28; *p* = 0.816). Intraoperative low EtCO_2_, specifically a mean intraoperative EtCO_2_ below 35 mmHg, was not substantially associated with either increased incidence of PONV or prolonged PLOS.

## Introduction

The incidence of postoperative nausea and vomiting (PONV) remains high despite considerable improvements in treatment over the past few decades. PONV is nausea or vomiting in the first 24–48 h after surgery^1^. Well-established risk factors for PONV include female gender, history of PONV or motion sickness, nonsmoking, and postoperative opioid use^2^. The risk of PONV is up to 80% in high-risk patients with all four risk factors^3^. The incidence of PONV is particularly high among patients undergoing gynecologic laparoscopic surgery^4^. PONV is associated with decreased patient satisfaction^5^, increased postoperative complications^6^, and longer postoperative length of hospital stay (PLOS) ^7^.

Hypocapnia may be associated with decreased systemic vasodilation^8^ and may cause tissue ischemia^9^, intestinal ischemia^10^, and cerebral ischemia^11,12^. Animal studies have reported that serotonin levels in the brain, a highly emetogenic substance, increase with intestinal ^13,14^ and cerebral ischemia^15^. Based on the hypothesis associating hypocapnia with increased serotonin levels due to intestinal and cerebral ischemia, studies associate intraoperative hypocapnia with increased incidence of PONV^16,17^. However, the relationship between hypocapnia and PONV remains unclear because some studies had conflicting results^18,19^.

Therefore, we examined the association between intraoperative end-tidal carbon dioxide (EtCO_2_) and the incidence of PONV in patients undergoing gynecologic laparoscopic surgery. We adjusted for important confounding factors and assessed the effects of the duration and severity of low EtCO_2_ exposure.

## Methods

### Ethics

The Certified Review Board of Kyoto University, Kyoto, Japan (Chairperson Prof. Shinji Kosugi) approved the protocol for this study (approval no.: R1272-3, January 23, 2020). Additionally, the informed consent requirement was waived due to this study’s retrospective nature.

### Study design, setting, and population

In this single-center retrospective cohort study, we used data from the Kyoto University Hospital IMProve Anaesthesia Care and ouTcomes (Kyoto-IMPACT) database. The Kyoto-IMPACT database aims to clarify the relationship between intraoperative respiratory and cardiovascular parameters and postoperative outcomes. We consecutively selected patients who underwent surgery under the care of anesthesiologists at Kyoto University Hospital (1121 beds). We have published several studies using the Kyoto-IMPACT database^20,21^. We included adult female patients aged 18 years or older who underwent gynecologic laparoscopic surgery (i.e., adnexal surgery and/or hysterectomy) at Kyoto University Hospital between January 2012 and December 2017. The gynecologic laparoscopic surgery population was selected because the predicted incidence rate of PONV in this population is 30–40%, assumed to be a medium risk of PONV^4^. The exclusion criteria were as follows: (1) patients with postoperative intensive care unit admission; (2) those who underwent multiple surgeries within one week during the study period; (3) those who received epidural anesthesia; (4) those with missing smoking data, and (5) those with missing intraoperative EtCO_2_ data.

### Data collection

We collected data from the anesthesia information management and electronic medical record systems and constructed the Kyoto-IMPACT database. EtCO_2_ was continuously measured using a sidestream gas analyzer (GF-220R Multigas/Flow Unit, Nihon Kohden^®^, Japan) that was automatically uploaded to the anesthesia information management system every 1960s. Intraoperative EtCO_2_ was the mean EtCO_2_ level from skin incision to skin closure. We removed EtCO_2_ levels lower than 20 mmHg as artifacts (EtCO_2_ during aspiration or position change). The definitions of variables, including the minimum and maximum EtCO_2_ levels, can be found in Supplementary Data Table S1. We collected data on PONV by reviewing all clinical data contained in the electronic medical records. Ward nurses assessed the presence of nausea and vomiting at least twice daily. We defined PONV as one or more episodes of nausea or vomiting during the first 2 days after surgery and vomiting as one or more episodes of vomiting during the same period.

### Exposure

To determine how EtCO_2_ affects PONV, we defined exposure by calculating the dose, time, and cumulative effects of EtCO_2_. First, we evaluated the dose effects of EtCO_2_ using the mean EtCO_2_. Next, we divided the patients into two groups based on the cutoff EtCO_2_ level of 35 mmHg proposed by Way and Hill^22^. We defined low EtCO_2_ as a mean EtCO_2_ lower than 35 mmHg and normal EtCO_2_ as a mean EtCO_2_ greater than or equal to 35 mmHg. We classified the patients in either of these groups and used them as the primary exposure for further analysis. Additionally, we categorized the mean EtCO_2_ levels into quartiles (i.e., < 35, 35–37, 37–40, and ≥ 40 mmHg) because the relationship between EtCO_2_ and PONV might not be linear. To assess the effects of the duration and severity of low EtCO_2_ exposure, we determined the time effect based on the minutes when the EtCO_2_ level was below 35 mmHg and measured the cumulative effect as the area with EtCO_2_ levels below the threshold of 35 mmHg for each patient. Furthermore, we categorized the minutes and area under the threshold of an EtCO_2_ level of 35 mmHg into quartiles; the lowest quartile was the reference category.

### Outcomes

The primary outcome in this study was PONV, defined as PONV for two days postoperatively. The secondary outcomes were nausea for two days postoperatively, vomiting for two days postoperatively, PONV for 3–7 days postoperatively, and PLOS. We defined PLOS as the duration of hospital stay after surgery for patients who survived until discharge.

### Statistical analysis

We analyzed the relationship between intraoperative EtCO_2_ and PONV before data collection. We used the Mann–Whitney test for group comparisons, and continuous variables were expressed as the median and interquartile range (IQR), and categorical variables were expressed as counts and percentages (%).

First, we performed modified Poisson regression analysis with robust variance to calculate the risk ratio for low EtCO_2_ (mean EtCO_2_ of less than 35 mmHg) and PONV, with the reference category of normal EtCO_2_ (mean EtCO_2_ ≥ 35 mmHg)^23^. Additionally, we calculated the risk ratios of the mean EtCO_2_ level in the first quartile (mean EtCO_2_ of less than 35 mmHg), third quartile (mean EtCO_2_ of 37–40 mmHg), and fourth quartile (mean EtCO_2_ of more than or equal to 40 mmHg). The second quartile (mean EtCO_2_ of 35–37 mmHg) was the reference category because it was considered normocapnia. Furthermore, we examined the time and cumulative effects of EtCO_2_ by evaluating how each quartile affected PONV, with the first quartile (with minutes under an EtCO_2_ of 35 mmHg and the area below the threshold of 35 mmHg) being the reference category. We created a model using the covariates previously used to demonstrate the relationship between intraoperative EtCO_2_ and PONV. The model included age, smoking history, surgery duration, body mass index (BMI), total intravenous anesthesia (TIVA), mean arterial pressure (MAP), intraoperative fentanyl use, postoperative fentanyl dose for intravenous patient-controlled analgesia (IVPCA), the use of prophylactic antiemetics, the addition of droperidol to postoperative IVPCA, American Society of Anesthesiologists Physical Status (ASAPS), malignancy, and emergency surgery. Additionally, a modified Poisson regression model investigated whether the dose, time, or cumulative effect of EtCO_2_ affects postoperative nausea two days, vomiting two days, and PONV 3–7 days postoperatively, adjusting for the aforementioned models. To further evaluate the relationship between EtCO_2_ and PLOS, we performed a linear regression analysis adjusting for the possible covariates in the aforementioned models.

The relationship between intraoperative EtCO_2_ and PONV may depend on patient and surgical characteristics. Therefore, we performed a subgroup analysis to assess this potential heterogeneity. We used the modified Poisson regression model for the following subgroups: (1) age (≥ 50/ < 50 years), (2) malignancy (yes/no), (3) smoking history (ever smoked/never smoked), (4) duration of surgery (≥ 4/ < 4 h), (5) TIVA (yes/no), (6) the use of intraoperative prophylactic antiemetics (yes/no), (7) postoperative fentanyl dose for IVPCA (≥ 20/ < 20 μg/h) and (8) addition of droperidol in IVPCA (yes/no). We calculated the crude risk ratio of PONV in each subgroup and examined the interaction between subgroups and the mean of intraoperative EtCO_2_.

To maximize statistical power, all eligible patients enrolled in the Kyoto-IMPACT database since 2012, when postoperative nausea and vomiting began to be recorded in their current form, were included in the analysis. To determine the study power, we estimated that approximately 120 laparoscopic gynecologic surgeries were performed annually at Kyoto University Hospital, with 720 surgeries performed over six years. The risk ratio was 1.53, the incidence of PONV was 40%^4^, and the proportion of low EtCO_2_ was 50%^24^, giving an estimated power of 80%. The rate of missing data was 0.04%, so we conducted a complete case analysis. All statistical tests were two-tailed. We used Stata/SE 15.1 (StataCorp LLC, College Station, Texas, USA) to conduct all statistical analyses.

### Ethics approval

All procedures performed in studies involving human participants were in accordance with the ethical standards of the institutional and/or national research committee and with the 1964 Helsinki Declaration and its later amendments or comparable ethical standards. The Certified Review Board of Kyoto University, Kyoto, Japan (Chairperson Prof. Shinji Kosugi) approved the protocol for this study (approval no.: R1272-3, January 23, 2020). Additionally, the informed consent requirement was waived due to this study’s retrospective nature.

## Results

### Baseline patient characteristics

**Figure 1:**
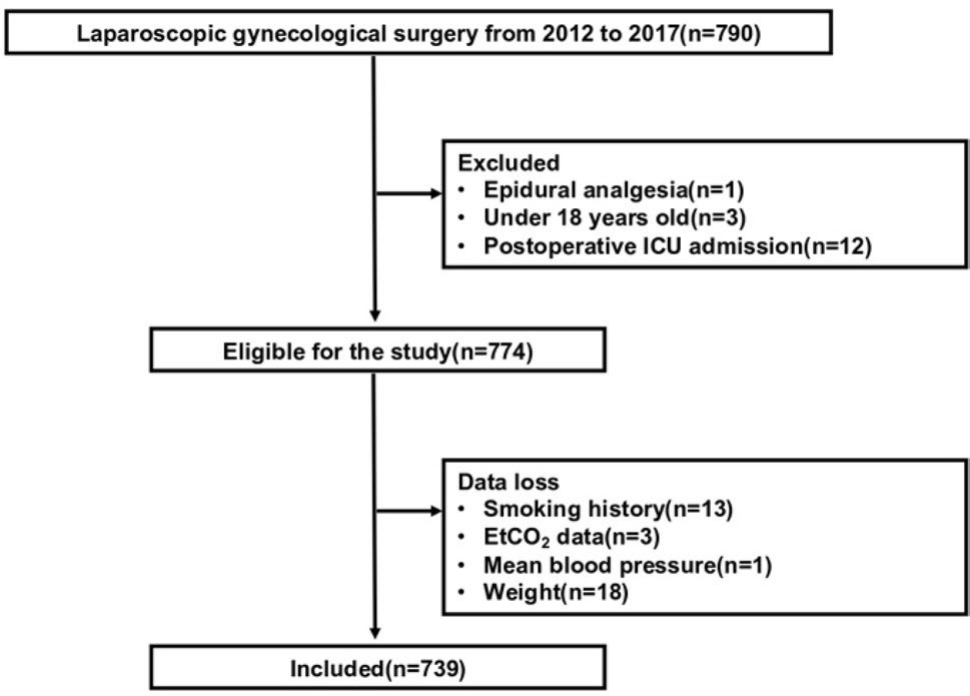
Flowchart of this study. We consecutively included patients aged 18 years or older who underwent laparoscopic gynecologic surgery under general anesthesia at Kyoto University Hospital from 2012 to 2017. Subsequently, cases that met the eligibility criteria were selected and analyzed as complete cases.

Of the 790 patients who underwent laparoscopic gynecologic surgery between 2008 and 2017, 774 met our inclusion criteria, and we included 739 in the complete case analysis (Fig. 1). Low EtCO_2_ (defined as the mean EtCO_2_ level of less than 35 mmHg) occurred in 120 patients (16%), whereas PONV occurred in 430 patients (58%). Table 1 shows the overall baseline characteristics of the study participants. The median EtCO_2_ values were 37 mmHg (IQR, 35–40 mmHg) overall, 33 mmHg (IQR, 32–34 mmHg) in patients with low EtCO_2_, and 38 mmHg (IQR, 36–40 mmHg) in patients with normal EtCO_2_.

**Table 1:** Patient characteristics (n = 739).

Characteristics	All patients (n = 739)	Low EtCO_2_ (n = 120)	Normal EtCO_2_ (n = 619)
Age (years)	45 (36–56)	47 (34–58)	44 (36–55)
**ASA-PS**			
I	402 (54.55%)	60 (50.42%)	342 (55.34%)
II	322 (43.69%)	58 (48.74%)	264 (42.72%)
III	13 (1.76%)	1 (0.84%)	12 (1.94%)
BMI	21.28 (19.35–23.62)	21.73 (19.38–24.45)	21.16 (19.35–23.52)
Malignant	205 (27.74%)	25 (20.83%)	180 (29.08%)
Never smoker	567 (76.73%)	87 (72.50%)	480 (77.54)
Emergency surgery	42 (5.70%)	6 (5.04%)	36 (5.83%)
Duration of surgery (min)	186 (125–270)	156 (110–233)	195 (129–276)
Blood loss (ml)	10 (0–100)	0 (0–75)	17 (0–100)
Transfusion volume (ml)	0 (0)	0 (0)	0 (0)
Infusion volume (ml)	1400 (1000–2040)	1265 (920–1920)	1450 (1000–2060)
TIVA	135 (18.27%)	25 (20.83%)	110 (17.77%)
Mean MAP (mmHg)	73 (68–80)	73 (68–81)	73 (68–80)
Intraoperative antiemetics use	284 (38.43%)	37 (30.83%)	247 (39.90%)
Addition of droperidol in IVPCA	321 (43.44%)	38 (31.67%)	283 (45.72%)
Total intraoperative fentanyl dose (μg)	200 (150–250)	200 (100–250)	200 (150–250)
Postoperative fentanyl dose in IVPCA (μg/h)	20 (0–25)	20 (0–25)	20 (0–25)
Mean EtCO_2_	37 (35–40)	33 (32–34)	38 (36–40)
Minimum EtCO_2_	31 (29–33)	28 (26–30)	32 (30–34)
Maximum EtCO_2_	42 (40–46)	37 (36–39)	43 (41–47)

Values are given as median (interquartile range) or count (%).

*ASAPS* American Society of Anesthesiologists Physical Status, *BMI* body mass index, *TIVA* total intravenous anesthesia, *MAP* mean arterial pressure, *IVPCA* intravenous patient-controlled analgesia, *EtCO*_*2*_ end-tidal carbon dioxide.

### Association between low EtCO_2_and PONV

Table 2 shows the study’s main results. PONV occurred in 67 (55.83%) of the 120 patients in the low EtCO_2_ group, whereas 363 (58.64%) of the 619 patients were in the normal EtCO_2_ group. We could not find a substantial association between low EtCO_2_ and PONV (crude risk ratio, 0.95; 95% confidence interval [CI] 0.80–1.13; *p* = 0.577) (adjusted risk ratio, 0.96; 95% CI 0.80–1.14; *p* = 0.658). For further analysis, we divided EtCO_2_ into quartiles. The second quartile (mean EtCO_2_ 35–37 mmHg) was the reference, and the definition of low EtCO_2_ was the lowest quartile of mean EtCO_2_ (mean EtCO_2_ of less than 35 mmHg). The second (mean EtCO_2_ of 35–37 mmHg), third (mean EtCO_2_ 37–40 mmHg), and fourth (mean EtCO_2_ ≥ 40 mmHg) quartiles of mean EtCO_2_ values were not substantially associated with increased incidence of PONV, with low EtCO_2_ (first quartile [mean EtCO_2_ of less than 35 mmHg]) as the reference category.

**Table 2:** Multivariable analysis of the relationship between EtCO_2_ and POD2-PONV.

	N	POD2-PONV	Crude risk ratio (95% CI)	*P*-value	Adjusted risk ratio (95% CI)	*P*-value
**Mean EtCO**_**2**_						
Normal EtCO_2_	619	363 (58.64%)	1	–	1	–
Low EtCO_2_	120	67 (55.83%)	0.95 (0.80–1.13)	0.577	0.96 (0.80–1.14)	0.658
**Mean EtCO**_**2**_						
< 35 mmHg	120	67 (55.83%)	1.01 (0.82–1.24)	0.906	1.04 (0.85–1.28)	0.650
35–37 mmHg	171	101 (59.06%)	1.07 (0.89–1.27)	0.451	1.09 (0.92–1.30)	0.284
37–40 mmHg	254	155 (61.02%)	1.10 (0.94–1.29)	0.217	1.15 (0.98–1.34)	0.079
≥ 40 mmHg	194	107 (55.15%)	1	–	1	–
**Minutes below EtCO**_**2**_** 35 mmHg**						
Quartile value 1 (0–11 min)	185	102 (55.14%)	1	–	1	–
Quartile value 2 (12–25 min)	187	106 (56.68%)	1.02 (0.85–1.23)	0.764	1.04 (0.87–1.24)	0.653
Quartile value 3 (26–66 min)	181	110 (60.77%)	1.10 (0.92–1.31)	0.276	1.10 (0.93–1.30)	0.222
Quartile value 4 (67–613 min)	186	112 (60.22%)	1.09 (0.91–1.30)	0.323	1.03 (0.87–1.22)	0.700
**Area under the threshold of EtCO**_**2**_** 35 mmHg**						
Quartile value 1 (0–7)	183	98 (53.55%)	1	–	1	–
Quartile value 2 (8–36)	182	104 (57.14%)	1.03 (0.86–1.23)	0.744	1.01 (0.85–1.21)	0.825
Quartile value 3 (37–107)	186	113 (60.75%)	1.08 (0.91–1.29)	0.358	1.10 (0.93–1.30)	0.232
Quartile value 4 (108–2213)	188	115 (61.17%)	1.08 (0.91–1.29)	0.346	1.03 (0.87–1.23)	0.654

*EtCO*_*2*_ end-tidal carbon dioxide, *POD 2* postoperative day 2, *PONV* postoperative nausea and vomiting, *CI* confidence interval.

For the time effects of EtCO_2_, compared with short-term exposure (first quartile of exposure time to EtCO_2_ of less than 35 mmHg, 0–11 min), long-term exposure to EtCO_2_ levels of less than 35 mmHg (fourth quartile of exposure time to EtCO_2_ of less than 35 mmHg, 67–613 min) was not substantially associated with increased incidence of PONV (crude risk ratio, 1.09; 95% CI 0.91–1.30; *p* = 0.323) (adjusted risk ratio, 1.03; 95% CI 0.87–1.22; *p* = 0.700).

Finally, for the cumulative effects of EtCO_2_, the fourth quartile of the area under the EtCO_2_ threshold of 35 mmHg (108–2213) was not substantially associated with increased incidence of PONV compared with the first quartile (0–7) (crude risk ratio, 1.08; 95% CI 0.91–1.29; *p* = 0.346) (adjusted risk ratio, 1.03; 95% CI 0.87–1.23; *p* = 0.654).

### Association between low EtCO_***2***_ and nausea and vomiting 2 days postoperatively and PONV 3–7 day postoperatively

The adjusted risk ratio for the low EtCO_2_ group (mean EtCO_2_ of less than 35 mmHg) did not indicate an association between low EtCO_2_ and nausea and vomiting two days postoperatively or PONV 3–7 days postoperatively (Table 3), with normal EtCO_2_ being the reference category.

**Table 3:** Multivariable analysis of the relationship between EtCO_2_ and secondary outcomes.

	Number of events (%)	Crude risk ratio (95% CI)	*P*-value	Adjusted risk ratio (95% CI)	*P*-value
**POD2: postoperative nausea**					
Normal EtCO_2_	346/619 (55.90%)	1	–	1	–
Low EtCO_2_	66/120 (55.00%)	0.98 (0.82–1.17)	0.857	0.99 (0.82–1.18)	0.916
**POD2: postoperative vomiting**					
Normal EtCO_2_	184/619 (29.73%)	1	–	1	–
Low EtCO_2_	37/120 (30.83%)	1.03 (0.77–1.39)	0.807	1.17 (0.88–1.55)	0.264
**POD 3–7: PONV**					
Normal EtCO_2_	383/619 (61.87%)	1	–	1	–
Low EtCO_2_	70/120 (58.33%)	0.94 (0.80–1.11)	0.480	0.95 (0.81–1.12)	0.583

*EtCO*_*2*_ end-tidal carbon dioxide, *POD 2* postoperative day 2, *POD 3–7* postoperative days 3 to 7, *PONV* postoperative nausea and vomiting, *CI* confidence interval.

### Association between low EtCO_***2***_ and PLOS

The median PLOS was 6 days (IQR, 5–8 days) (Table 4). The median PLOS in patients with low EtCO_2_ was not different from that in patients with normal EtCO_2_ (6 days [IQR, 5–8 days] vs. 6 days (IQR, 5–7 days); *p* = 0.782). Linear regression analysis showed that low EtCO_2_ was not likely to be associated with PLOS (crude adjusted difference in PLOS, − 0.15; 95% CI − 1.29 to 0.97; *p* = 0.783) (adjusted difference in PLOS, − 0.13; 95% CI − 1.00 to 1.28; *p* = 0.816).

**Table 4:** Multivariable analysis of the relationship between EtCO_2_ and PLOS.

	Median (IQR)	P value	Crude difference in PLOS (95% CI)	P-value	Adjusted difference in PLOS (95% CI)	*P*-value
**Length of stay(day)**						
Normal EtCO_2_	6 (5–8)	0.782	1	–	1	–
Low EtCO_2_	6 (5–7)		− 0.15 (− 1.29 to 0.97)	0.783	0.13 (− 1.00 to 1.28)	0.816

*EtCO*_*2*_ end-tidal carbon dioxide, *PLOS* postoperative length of stay, *IQR* interquartile range, *CI* confidence interval.

### Subgroup analysis

Subgroup analyses included age (≥ 50/< 50 years), malignancy, smoking history, duration of surgery (≥ 4 h/< 4 h), TIVA, the use of intraoperative prophylactic antiemetics, postoperative fentanyl dose for IVPCA (≥ 20 μg/h/< 20 μg/h) and addition of droperidol in IVPCA. There was no interaction between these variables and PONV (Table 5).

**Table 5:** Subgroup analyses stratified by patient and operative variable.

	N	POD2-PONV	Crude risk ratio (95% CI) of low EtCO_2_	*P*-value	P for interaction
Overall	739	430 (58.19%)	0.95 (0.80–1.13)	0.577	
**Age (year)**					0.837
< 50	454	246 (54.19%)	0.96 (0.76–1.20)	0.725	
≥ 50	285	184 (64.56%)	0.91 (0.70–1.18)	0.486	
**Malignancy**					0.594
Yes	205	135 (65.85%)	0.90 (0.64–1.26)	0.540	
No	534	295 (55.24%)	0.98 (0.80–1.20)	0.913	
**Smoking history**					0.640
Ever	172	92 (53.49%)	1.02 (0.72–1.45)	0.892	
Never	567	338 (59.61%)	0.93 (0.76–1.14)	0.511	
**Duration of surgery (h)**					0.491
≥ 4	238	148 (62.18%)	0.87 (0.61–1.23)	0.442	
< 4	501	282 (56.29%)	0.99 (0.51–1.21)	0.959	
**TIVA**					0.274
Yes	604	369 (61.09%)	0.91 (0.76–1.10)	0.376	
No	135	61 (45.19%)	1.19 (0.77–1.83)	0.428	
**Intraoperative antiemetics**					0.990
Yes	284	168 (59.15%)	0.95 (0.70–1.28)	0.757	
No	455	262 (57.58%)	0.95 (0.77–1.17)	0.666	
**Postoperative fentanyl dose in IVPCA (μg/h)**					0.921
< 20	246	121 (49.19%)	0.99 (0.73–1.35)	< 20	
≥ 20	493	309 (62.68%)	0.98 (0.80–1.21)	≥ 20	
**Addition of droperidol in IVPCA**					0.502
Yes	321	175 (54.52%)	1.01 (0.75–1.37)	Yes	321
No	418	255 (61.00%)	0.90 (0.73–1.11)	No	418

*POD 2* postoperative day 2, *PONV* postoperative nausea and vomiting, *CI* confidence interval, *TIVA* total intravenous anesthesia, *IVPCA* intravenous patient-controlled analgesia.

## Discussion

In this retrospective cohort study, mean of intraoperative EtCO_2_ was not substantially associated with increased incidence of PONV and prolonged PLOS in patients undergoing gynecologic laparoscopic surgery. Furthermore, we examined the effects of the duration and severity of low EtCO_2_ exposure using the time and cumulative effects of EtCO_2_ but found no clear association.

Two small studies have studied whether there is an association between low EtCO_2_ and PONV^17,18^, but the results have been inconsistent. A randomized controlled trial (RCT) involving 75 patients who underwent percutaneous nephrolithotripsy reported that the hypercapnia management group had less PONV^17^. However, a prospective observational study involving 90 pediatric patients who underwent inguinal surgery has reported that elevated levels of EtCO_2_ were an independent predictor of PONV^18^. As the aforementioned studies have different types of surgery and patient backgrounds, their results might not be directly applicable to patients undergoing gynecologic laparoscopic surgery.

Furthermore, three studies on patients who had undergone gynecologic surgery have shown inconsistent results. An RCT involving 387 patients who underwent gynecologic laparoscopic surgery reported mild hypercapnia management did not reduce PONV^19^. That study did not evaluate the effects of low EtCO_2_ (mean EtCO_2_ level of less than 35 mmHg). Alternatively, a retrospective cohort study involving 146 patients undergoing open gynecologic surgery has reported that the minimum EtCO_2_ level of ≤ 31 mmHg lasting longer than 10 min was associated with an increased incidence of PONV^16^. Still, that study only evaluated the effects of extremely low EtCO2 levels (mean EtCO2 of ≤ 31 mmHg). It did not evaluate the dose and time effects of low EtCO2 below the commonly defined EtCO_2_ level of 35 mmHg. Furthermore, an RCT involving 60 patients undergoing gynecologic laparoscopic surgery reported that low EtCO_2_ management reduced the incidence of nausea, PONV score, and the use of rescue antiemetics^25^; these results differed from the two aforementioned studies. Management to keep EtCO2 at a low level may avoid PONV by inhibiting cerebral vasodilation, preventing increased intracranial pressure caused by the pneumoperitoneum and Trendelenburg position, which would not affect the ischemia-sensitive vestibular system. However, this study may have an internal validity problem in which it was not blinded. Furthermore, it had a generalizability problem because it excluded patients with severe systemic diseases, ASAPS-III patients, those with a history of PONV motion sickness, and smokers.

Considering that the results of previous studies are inconsistent, the evidence on the association between intraoperative low EtCO_2_ and PONV remains limited. Therefore, we conducted this study, which involved the largest cohort from real-world data, which provided a sufficient sample size, resulting in a statistical power of 80% to detect a risk ratio of 1.53. Furthermore, adjusting for important confounders, such as blood pressure, age, and intraoperative fentanyl use, and assessing the dose–effect of low EtCO_2_ (mean EtCO_2_ of less than 35 mmHg) and the effects of the duration and severity of low EtCO_2_ exposure, we could not demonstrate an association between low EtCO_2_ and PONV. Even extremely low EtCO_2_, defined as EtCO_2_ of less than 31 mmHg sustained for more than 10 min^16^, failed to show an association with PONV (Supplemental Data Table S2).

This study has several strengths. First, it investigated the association between the effects of EtCO_2_ and PONV and PLOS, the dose effects of EtCO_2_ (mean level of less than 35 mmHg) and the effects of the duration (time effects, long-term exposure to EtCO_2_ of less than 35 mmHg) and severity (cumulative effects, area under the threshold of EtCO_2_ of less than 35 mmHg). Among the three previous studies that examined the association between intraoperative low EtCO_2_ and PONV, which only evaluated the dose effects ^17–19^, only one study evaluated the association between the time effects of low EtCO_2_ and PONV^16^. Second, this study adjusted for potential confounding factors that were not adjusted in previous studies, such as blood pressure, age, and intraoperative fentanyl use, using a modified Poisson regression model. Third, this was a large study with sufficient sample size. All previous studies had small sample sizes, so the number of confounding factors that can be adjusted is limited.

This study has several limitations. First, we extracted information on the presence of nausea and vomiting from the records of assessments performed by the ward nurses at least twice a day, so PONV occurring at other times may have been overlooked. However, we thought that moderate to severe PONV reported voluntarily by patients or required treatment was fully measured. Second, we did not consider the PaCO_2_–EtCO_2_ gap to calibrate EtCO_2_ levels using PaCO_2_ levels. Thus, we underestimated the effects of low EtCO_2_ and overestimated the effects of hypercapnia. However, since PaCO_2_ is usually 2–5 mmHg higher than EtCO_2_ in healthy populations, this was considered a limited effect. Last, there may be unknown and unmeasured confounding factors, such as potential reasons for anesthesiologists to target a specific EtCO_2_ level, missing data on intraoperative ventilation parameters, and PONV risk factors among patient factors is, history of PONV and motion sickness.

## Conclusion

Intraoperative low EtCO_2_ (mean EtCO_2_ level less than 35 mmHg) was not substantially associated with either increased incidence of PONV or prolonged PLOS in patients undergoing gynecologic laparoscopic surgery.

## Supplementary Information

Supplementary Information.
