# Supplementary material for: Association between intraoperative end-tidal carbon dioxide and postoperative nausea and vomiting in gynecologic laparoscopic surgery
Source: Sci Rep. 2022 Apr 27;12:6865. doi: 10.1038/s41598-022-10727-6 (PMC9046149; doi:10.1038/s41598-022-10727-6)
Supplement: Supplementary file 1 — Supplementary Information. [file 41598_2022_10727_MOESM1_ESM.pdf]

**Supplemental data**

**Table S1. Definitions of variables**

| Variables | Definitions |
|---|---|
| Age | In years, recorded in anesthesia information management system |
| ASAPS | 1 to 4, recorded in anesthesia information management system |
| BMI | the body mass divided by the square of the body height, recorded in the anesthesia information management system |
| Malignant | Preoperative tumor classification (benign or malignant), recorded in the anesthesia information management system |
| Never smoker | Smoking history recorded in the anesthesia information management system |
| Emergency surgery | An emergency procedure, recorded in anesthesia information management system |
| Epidural anesthesia | Defined as an epidural catheter placement documented in anesthesia information management system, regardless of the duration of catheter placement, recorded in anesthesia information management system |
| Duration of surgery | In hours, from skin incision to skin closure, recorded in anesthesia information management system |
| Blood loss | In mL, recorded in anesthesia information management system |
| Transfusion volume | In mL, recorded in anesthesia information management system of administration of red blood cells, fresh frozen plasma and platelet during surgery |
| Infusion volume | In mL, recorded in anesthesia information management system |

| | |
|---|---|
| TIVA | Anesthesia method (inhalation anesthesia or TIV), recorded in the anesthesia information management system |
| Mean MAP | Mean MAP was calculated by dividing the total intraoperative MAP by the duration from skin incision to skin closure for each patient. Intraoperative MAP, which was defined as MAP from skin incision to skin closure extracted from the anesthesia information management system |
| Intraoperative antiemetics use | Use of intraoperative antiemetics (droperidol, metoclopramide, steroids) recorded in the anesthesia information management system |
| Addition of droperidol in IVPCA | Use of droperidol for postoperative IVPCA, recorded in the anesthesia information management system |
| Total intraoperative fentanyl dose (µg) | Total intraoperative fentanyl dose, recorded in the anesthesia information management system |
| Postoperative fentanyl dose in IVPCA (µg/h) | Postoperative fentanyl dose in IVPCA, recorded in the anesthesia information management system |
| Mean EtCO2 | Defined by dividing the total intraoperative EtCO2 from skin incision to skin closure for each patient |
| Minimum EtCO2 | Defined as minimum EtCO2 >20 mmHg from skin incision to skin closure extracted from the anesthesia information management system |
| Maximum EtCO2 | Defined as maximum EtCO2 from skin incision to skin closure, extracted from the anesthesia information management system |
| Length of hospital stay | The duration of hospitalization, among patients who survived hospital discharge |

ASAPS, the American Society of Anesthesiologists physical status; BMI, Body Mass Index; COPD, Chronic Obstructive Pulmonary Disease; MAP, Mean arterial pressure; ICD-10, International Classification of Diseases, Tenth Revision; EtCO2, end-tidal carbon dioxide

23.9% of patients with laparoscopic gynecological surgery used arterial catheters, recording MAP every minute. The other patients used non-invasive blood pressure monitoring, recording MAPs every 2–5 min. If no arterial catheter was used, non-invasive blood pressure was substituted. We also used the MAP artefact removal algorithm, referenced in the previous study[1]: (1) out-of-range pressures: (a) SBP (systolic blood pressure) ≥ 300 or SBP ≤ 0 mmHg, (b) SBP ≤ DBP (diastolic blood pressure) + 5 mmHg or (c) DBP ≤ 0 mmHg or DBP ≥ 225 mmHg; (2) sudden change in SBP ≥ 80 mmHg within 1 min.

**Table S2. Multivariable analysis of the relationship between EtCO2 and PONV.**

| | POD2-PONV(%) | Crude Risk Ratio (95% CI) | *P*-value | Adjusted Risk Ratio (95% CI) | *P*-value |
|---|---|---|---|---|---|
| **Mean EtCO2** | | | | | |
| <31mmHg | 9/15(60.00%) | 1.03(0.67-1.56) | 0.883 | 0.96(0.63-1.46) | 0.857 |
| ≥31mmHg | 421/724(58.15%) | 1 | - | 1 | - |
| **Minutes below EtCO2 31mmHg** | | | | | |
| short duration(< 10 min) | 312/544(57.35%) | 1 | - | 1 | - |
| long duration(≥10min) | 118/195(60.51%) | 1.05(0.92-1.20) | 0.435 | 1.03(0.90-1.17) | 0.645 |

Abbreviations: EtCO2, end-tidal carbon dioxide; POD, postoperative day; PONV, postoperative nausea and vomiting; CI, confidence interval.

Reference:


1. Salmasi V, Maheshwari K, Yang D, et al. Relationship between Intraoperative Hypotension, Defined by Either Reduction from Baseline or Absolute Thresholds, and Acute Kidney and Myocardial Injury after Noncardiac Surgery: A Retrospective Cohort Analysis. Anesthesiology.2017; **126**: 47–65.
